# The Effects of Sodium Dichloroacetate on Mitochondrial Dysfunction and Neuronal Death Following Hypoglycemia-Induced Injury

**DOI:** 10.3390/cells8050405

**Published:** 2019-05-01

**Authors:** A Ra Kho, Bo Young Choi, Song Hee Lee, Dae Ki Hong, Jeong Hyun Jeong, Beom Seok Kang, Dong Hyeon Kang, Kyoung-Ha Park, Jae Bong Park, Sang Won Suh

**Affiliations:** 1 Department of Physiology, College of Medicine, Hallym University, Chuncheon 24252, Kangwon-Do, Korea; rnlduadkfk136@hallym.ac.kr (A.R.K.); bychoi@hallym.ac.kr (B.Y.C.); sshlee@hallym.ac.kr (S.H.L.); zxnm01220@gmail.com (D.K.H.); jd1422@hanmail.net (J.H.J.); ttiger1993@gmail.com (B.S.K.); 2 Department of Medical science, College of Medicine, Hallym University, Chuncheon 24252, Kangwon-Do, Korea; ehdgus6312@gmail.com; 3 Division of Cardiovascular Disease, Hallym University Medical Center, Anyang 14068, Korea; pkhmd@naver.com; 4 Department of Biochemistry, College of Medicine, Hallym University, Chuncheon 24252, Kangwon-Do, Korea; jbpark@hallym.ac.kr

**Keywords:** hypoglycemia, sodium dichloroacetate, pyruvate dehydrogenase kinase, pyruvate dehydrogenase, oxidative stress, neuron death

## Abstract

Our previous studies demonstrated that some degree of neuronal death is caused by hypoglycemia, but a subsequent and more severe wave of neuronal cell death occurs due to glucose reperfusion, which results from the rapid restoration of low blood glucose levels. Mitochondrial dysfunction caused by hypoglycemia leads to increased levels of pyruvate dehydrogenase kinase (PDK) and suppresses the formation of ATP by inhibiting pyruvate dehydrogenase (PDH) activation, which can convert pyruvate into acetyl-coenzyme A (acetyl-CoA). Sodium dichloroacetate (DCA) is a PDK inhibitor and activates PDH, the gatekeeper of glucose oxidation. However, no studies about the effect of DCA on hypoglycemia have been published. In the present study, we hypothesized that DCA treatment could reduce neuronal death through improvement of glycolysis and prevention of reactive oxygen species production after hypoglycemia. To test this, we used an animal model of insulin-induced hypoglycemia and injected DCA (100 mg/kg, i.v., two days) following hypoglycemic insult. Histological evaluation was performed one week after hypoglycemia. DCA treatment reduced hypoglycemia-induced oxidative stress, microglial activation, blood–brain barrier disruption, and neuronal death compared to the vehicle-treated hypoglycemia group. Therefore, our findings suggest that DCA may have the therapeutic potential to reduce hippocampal neuronal death after hypoglycemia.

## 1. Introduction

Hypoglycemia is a potentially serious condition that occurs when blood glucose levels quickly fall below a specific threshold concentration. The most common cause of hypoglycemia is misuse of medications such as insulin and sulfonylureas to control blood glucose levels [1,2]. Medications that cause blood glucose levels to fall sharply or repeatedly can lead to hypoglycemic shock. In these cases, the first treatment for patients who present at the hospital in a hypoglycemic state of shock is to rapidly raise blood glucose levels, which is essential for resuscitating the patient and impossible to avoid. Clinically, patients display decreased cognitive abilities over time following such episodes [3], and our previous paper revealed that this condition causes an increase in neuronal cell death [4]. Therefore, this study is important to identify means to minimize neuronal cell death caused by hypoglycemia and glucose reperfusion, which is performed as an essential step in treating hypoglycemia.

Therefore, type 1 and type 2 diabetic patients are at an increased risk of experiencing hypoglycemia. Hypoglycemia is classified into three categories depending on the level of blood glucose: mild, moderate, and severe. Mild hypoglycemia occurs when the concentration of blood glucose falls below 70 mg/dL, leading to symptoms such as headache, sweating, and irritation. Moderate hypoglycemia (below 55 mg/dL) is more severe; thus, people need to regulate this condition carefully. The most serious form of hypoglycemia occurs when blood glucose levels fall below 35 mg/dL. Severe hypoglycemia can lead to seizure, loss of consciousness, and death in the most extreme cases. Previous studies demonstrated that some degree of the observed neuronal death is caused by hypoglycemia itself, but glucose reperfusion to recover the patient from critically low glucose levels acts to drive secondary injury, thereby inducing a second, more severe wave of neuronal death [5,6]. This process is called “glucose-reperfusion injury” after hypoglycemia by our laboratory [4,7]. This injury promotes an imbalance in several cellular programs that can cause neuronal cell death, including the production of reactive oxygen species (ROS), destruction of the blood–brain barrier (BBB), microglial activation associated with inflammation [8], and excessive zinc release [9].

The enzyme pyruvate dehydrogenase (PDH) has three main components: E1, decarboxylates pyruvate and acetylates lipoic acid; E2, dihydrolipoamide acetyltransferase, which uses covalently bound lipoic acid; and lipoic acid is reoxidized by E3, dihydrolipoyl dehydrogenase. In addition, there are other subunits, the E3 binding protein and two complex-controlling enzymes: PDH kinases (PDKs), which inactivate PDH, and PDH phosphatase, which reactivates the PDH. PDH is a key enzyme that catalyzes the oxidative decarboxylation of pyruvate to form acetyl-coenzyme A (acetyl-CoA) under normal conditions. PDH controls the influx of pyruvate into the mitochondria to initiate oxidative metabolism and is an important regulator of the citric acid cycle [10,11]. PDH dysfunction or deficiency most often arise due to mutations or inactivation by PDKs via E1, which causes malfunction of the citric acid cycle due to PDH inactivation and leads to cell death [12]. Previous studies reported that starvation and diabetes lead to enhanced PDK activity, leading to decreased activity of PDH [13,14,15,16,17,18]. This decreased PDH activity results from increased PDK expression. Starvation increases the level of PDK2 expression in liver and kidney [19].

PDKs have four isoforms: PDK1, PDK2, PDK3, and PDK4. PDK1 is expressed in the heart, pancreatic islet cells, and in muscles [20,21,22]. PDK3 is only been found in the testis and kidneys in small quantities [23]. PDK4 expression was observed in the heart, skeletal muscles, liver, and brain [19,22,24]. PDK2 is the most abundant isoenzyme in the rat brain [23], with PDK4 hardly being expressed under basal physiological conditions. PDKs phosphorylate the E1 subunit, thus inactivating PDH [25].

In general, previous studies showed that PDK inhibits PDH via phosphorylating PDH after brain injuries, such as ischemia or epilepsy, thereby restricting the pyruvate oxidation (PO) pathway available to brain cells. As a result, under this pathological condition, the citric acid cycle cannot proceed and the production of ATP decreases, leading to cell death [26,27].

Sodium dichloroacetate (DCA) has been studied for a long time, especially in cancer research. DCA, a known activator of PDH and inhibitor of PDK, is a mitochondria-targeting small molecule that can penetrate most tissues even after oral administration [28]. It is responsible for maintaining PDH in the dephosphorylated active form and improving the oxidation of pyruvate via inhibition of PDK [29]. DCA has been shown to instigate inhibition of fatty acid oxidation, which eventually can lead to an increase in acetyl-CoA concentration [30]. DCA stimulates glucose and lactate oxidation, thereby improving recovery of injury after ischemia [31,32,33]. Although DCA has toxic side effects in high doses, it has highly beneficial effects on outcomes for many diseases such as ischemia and cardiac arrest [34,35].

In our laboratory, we have suggested a number of possible methods for aiding in the prevention of hippocampal neuron death after hypoglycemia [4,8,9,36,37,38,39]. In our previous studies, we revealed that administration of pyruvate reduced severe hypoglycemia-induced neuronal cell death and cognitive impairment [37]. Therefore, we questioned whether supplementation of DCA (100 mg/kg, i.v., two days) could improve the oxidation of pyruvate and reduce severe hypoglycemia-induced neuronal cell death.

We found that DCA treatment inhibited PDK and significantly reduced oxidative stress, microglial activation, BBB disruption, and thus hippocampal neuronal death after severe hypoglycemia via enhancing PDH activation.

## 2. Materials and Methods

### 2.1. Ethics Statement

This study was conducted in strict accordance with the recommendation of the Institutional Animal Studies Care and Use Committee of the Hallym University in Chuncheon, Korea (Protocol # Hallym-2017-3). Animal sacrifice was conducted using isoflurane anesthesia and all attempts were made to minimize pain or distress.

### 2.2. Experimental Animals

We used adult male Sprague-Dawley rats (8 weeks old, 250–350 g, DBL Co., Eumseong, Korea). We accommodated the animals in a continuous temperature- and humidity-controlled environment (22 ± 2 °C, 55 ± 5% humidity, and a 12-h light:12-h dark cycle), and fed them with Purina diet (Purina, Gyeonggi, Korea) and water, both provided ad libitum. This study was written in accordance with the Animal Research: Reporting in Vivo Experiments (ARRIVE) guidelines.

### 2.3. Animal Surgery and Severe Hypoglycemia Induction

We induced hypoglycemia (HG) using human insulin [5,36]. Before inducing hypoglycemia, rats were fasted for 15–16 h. The next day, we performed intraperitoneal injection of 10 U/kg regular insulin (Novolin-R, Novo Nordisk, Bagsværd, Denmark). We conducted hypoglycemia surgeries under controlled ventilation with a small rodent respirator (Harvard Apparatus, South Natick, MA, USA). We inserted a catheter into the femoral artery to continuously monitor the arterial blood pressure and into the femoral vein for glucose infusion after hypoglycemia. To monitor electroencephalogram (EEG) signals, we pierced a small hole in the skull bilaterally and then inserted a reference needle in the neck muscle. We measured the blood pressure and EEG using the BIOPAC System (BIOPAC System Inc., Santa Barbara, CA, USA) in succession. We maintained the core temperature of animals at 36.5–37.5 °C and measured blood glucose levels at 30-min intervals to monitor induction of hypoglycemia.

When insulin reduces blood glucose levels to a level low enough to induce hypoglycemia, an isoelectric state is observed. Arterial blood pressure was sustained between 160 and 200 mmHg during the entire EEG isoelectric period. The severity of hypoglycemia-induced neuronal cell death is strongly related to the duration of the isoelectric (iso-EEG) period. We abruptly ended the hypoglycemic state at 30 min from initiation of iso-EEG [5,36] by suppling 25% glucose solution intravenously for 2 h (2.5 mL/h, i.v.) to recover normal blood glucose levels.

### 2.4. Injection of DCA

To ascertain the effect of DCA on hypoglycemia-induced neuronal death, the experimental group was divided into 4 groups: Sham (Vehicle, DCA) and Hypoglycemia (Vehicle, DCA). The control group was injected with 0.9% normal saline. Following hypoglycemia, animals were treated with DCA (100 mg/kg, i.v.) once a day for 2 days.

### 2.5. Brain Sample Preparation

To estimate the neuroprotective effects of DCA, we sacrificed animals 1 week after injury. Animals were deeply anesthetized with urethane injection (1.5 g/kg, i.p.). Brains were transcardially perfused with 500–600 mL of 0.9% saline and this was followed by 600 mL of 4% formaldehyde (FA). After perfusion, we removed the brain and then proceeded to post-fixation in the same fixative solution for roughly 1 h. After that, brains were immersed for cryoprotection in 30% sucrose for 2–3 days until the samples sank to the bottom. After cryoprotection, brain samples were frozen using freezing medium and were cut on a cryostat into 30-μm thicknesses and then kept in storage solution.

### 2.6. Detection of PDH and PDK2

To confirm PDH and PDK2 levels, we performed PDH and PDK2 staining with primary antibodies for each. Immunohistochemistry with PDH and PDK2 antibodies (Abcam, Cambridge, UK) was performed. Brain tissues were incubated in monoclonal rabbit antibody to rat PDH (diluted 1:100, ab168379) and a monoclonal rabbit antibody to rat PDK2 (diluted 1:100, ab68164) overnight in a 4 °C incubator. After washing, the sections were immersed in secondary antibody (PDH: Alexa Fluor 594-conjugated donkey anti-rabbit IgG secondary antibody, PDK2: Alexa Fluor 488-conjugated donkey anti-rabbit IgG secondary antibody, respectively, both diluted 1:250; Molecular Probes, Invitrogen, Carlsbad, CA, USA) for 2 h at room temperature (RT). The brain tissues were placed on gelatin-coated slides for observation under a microscope. To check the intensity of fluorescence of PDH and PDK2, we used ImageJ (version 1.47c; NIH, Bethesda, MD, USA) and the following steps were executed: select 100 cells in one brain tissue and then click the menu option Analyze → Measurement. (magnification = 80×).

### 2.7. Detection of Neuronal Death

To investigate neuronal death, brain samples were cut into 30 μm thicknesses on gelatin-coated slides for staining (Fisher Scientific, Pittsburgh, PA, USA). Fluoro-Jade B (FJB) staining was performed as descried by Hopkins and Schmued [40]. First, the slides were immersed in a basic alcohol solution (100→70%) and then washed with distilled water. After that, the slides were submerged in 0.06% potassium permanganate for 15 min. Secondly, the slides were dipped in 0.001% Fluoro-Jade B (Histo-Chem Inc., Jefferson AR, USA) for 30 min and washed 3 times for 10 min each in distilled water. After drying, we mounted a cover glass on each slide with a mixture of distyrene, a plasticizer, and xylene (DPX) solution and then observed the signals with a fluorescence microscope using blue (450–490 nm) excitation light. To quantify the result, we selected five coronal brain sections that were gathered from each animal starting 4.0 mm posterior to Bregma and collecting 5 sections at 75 μm intervals. A blind researcher counted the number of degenerating neurons in the same scope (magnification = 10×) of the hippocampal subiculum (900 × 1200 μm), CA1 (900 × 400 μm), and dentate gyrus (900 × 1200 μm) from both hemispheres. The total average number of degenerating neurons from each region was used for statistical analysis.

### 2.8. Detection of Live Neurons

To determine the number of live neurons present, we performed NeuN staining with a monoclonal anti-NeuN, clone A60 antibody (diluted 1:500, EMD Millipore, Billerica, MA, USA). We used it as the primary antibody in PBS with 30% Triton X-100 overnight in a 4 °C incubator. After washing three times for 10 min with PBS, and then incubating in anti-mouse IgG secondary antibody (diluted 1:250, Vector Laboraorise, Burlingame, CA, USA) for 2 h at RT, then, the samples were washed again. Next, the brain samples were put into the ABC solution (Burlingame, vector, CA, USA) for 2 h at RT on the shaker. After that, the samples were washed repeatedly three time for 10 min. To activate immune responses, we put samples in 0.01 M PBS buffer (100 mL) with 3,30-diaminobenzidine (0.06% DAB agar, Sigma-Aldrich Co., St. Louis, MO, USA) and 30% H₂O₂ (50 μL) for 1 min. After mounting the sample on the slides, we then used an Axioscope microscope to analyze the immunoreaction. A blinded experimenter counted the number of live neurons in a constant area (magnification = 10×) of the hippocampal subiculum (900 × 1200 μm), CA1 (900 × 400 μm), and dentate gyrus (900 × 1200 μm) from both hemispheres. The total average number of live neurons from each region was used for statistical analysis.

### 2.9. Detection of Oxidative Injury

To estimate oxidative injury after hypoglycemia, we conducted immunofluorescence staining with 4-hydroxy-2-nonenal (4HNE) antibodies (diluted 1:500, Alpha Diagnostic Intl. Inc., San Antonio, TX, USA) as described previously [4]. Brain sections were incubated in a mixture of polyclonal rabbit anti-HNE anti-serum and phosphate buffered saline (PBS) containing 0.3% TritonX-100 overnight in a 4 °C incubator. After washing three times for 10 min each with PBS, samples were also immersed in a mixture of Alexa Fluor 594-conjugated goat-anti rabbit IgG secondary antibody (diluted 1:250, Invitrogen, Grand Island, NY, USA) for two hours at room temperature. Finally, the sections were placed on gelatin-coated slides. To measure the oxidative stress signals, we used ImageJ using the following order of commands: click the menu option Image → Adjust → Color threshold and dark background. Then, we modulated brightness according to the intensity of oxidative stress. The image was converted to 8-bit images (magnification = 10×). The fluorescence intensity was expressed as mean gray value.

### 2.10. Detection of Microglia and Astrocyte Activation

To semi-quantitatively measure inflammation after injury, we performed CD11b and GFAP staining, a well-described indicator of the inflammatory response. Immunohistochemical staining was conducted with a mouse antibody to rat CD11b (diluted 1:500; AbD Serotec, Raleigh, NC, USA) and of goat antibody to rat GFAP (diluted 1:1000; Abcam, Cambridge, UK) in PBS containing 0.3% TritonX-100 overnight in a 4 °C incubator. After rinsing, the sections were immersed in Alexa Fluor 488-conjugated donkey anti-mouse IgG secondary antibody and Alexa Fluor 594-conjugated donkey anti-goat IgG secondary antibody (both diluted 1:250; Molecular Probes, Invitrogen) for two hours at RT. A randomly blinded experimenter then measured the intensity of astrocytes in the CA1 region. In the case of microglia, we used five sections from each animal to estimate scoring within the same CA1 region (magnification = 20×). To quantify microglial activation, a randomly blinded observer performed the measurements. Microglial activation criteria based on the number and intensity of CD11b positive cells and on their morphology are as follows [41,42]. The scores for each criterion are as follows: for *CD11b-positive cells number*, a score of 0 denotes no cells with continuously stained processes are present, score 1 denotes 1–9 cells with continuous processes per 100 μm^2^, score of 2 denotes 10–20 cells with continuous processes per 100 μm^2^, and score of 3 denotes >20 cells with continuous processes per 100 μm^2^; for *Intensity*, a score of 0 denotes no expression, 1 denotes weak expression, 2 denotes average expression, and a score of 3 denotes intense expression. When ImageJ is used, based on the max value 225, a score of 1 has intensity to mean value 1–50, a score of 2 has intensity to 51–100 and the last score of 3 has intensity to 100–max value 225.; and for *Morphology*, a score of 0 denotes 0% have activated morphology (amoeboid morphology with enlarged soma and thickened processes), 1 denotes 1–45% of microglia have the activated morphology, 2 denotes 45–90% of microglia, and a score of 3 denotes >90% of microglia have the activated morphology. Therefore, the total score includes the combination of three individual scores (CD11b-immunoreactive cell number, morphology and intensity), ranging from 0 to 9 (magnification = 20×).

### 2.11. Detection of BBB Disruption and Neutrophils

To check the degree of BBB disruption, we evaluated leakage of endogenous serum IgG after hypoglycemia. [43]. We conducted IgG staining with anti-rat IgG, which detects serum IgG released when the BBB is disrupted. Brain sections were incubated in a mixture of anti-rat IgG serum (diluted 1:250, Burlingame, Vector, CA, USA) in PBS containing 0.3% TritonX-100 on a shaking shelf for 2 hours at RT. After rinsing three times for 10 min each with PBS, we treated ABC solution for two hours at RT on a shaker. The ABC immunoperoxidase was used to reveal IgG-like immunoreactivity [44]. After that, the sections were exposed to 3,3′-diaminobenzidine (DAB ager, Sigma-Aldrich Co., St. Louis, MO, USA) in 0.1 M PBS buffer to trigger immune responses. After mounting, we observed the immune responses using an Axioscope microscope (Carl Zeiss, Munich, Germany) and quantified extravasation of endogenous serum IgG in the whole brain using the Image J program. The procedure for measuring IgG is as follows: Click the menu option Image→Type→8 bits and then, edit→invert. To measure the area, the menu option Analyze→Measure was selected. Mean gray values (magnification = 4×) were used. In addition, to measure the degree of BBB disruption, we evaluated any influx of neutrophils after hypoglycemia. We conducted myeloperoxidase (MPO) staining with rabbit anti-MPO (diluted 1:100, Invitrogen, Grand Island, NY, USA). The brain sections were soaked in the primary antibody solution with PBS containing 0.3% TritonX-100 overnight in a 4 °C incubator. After washing, samples were immersed in a mixture of Alexa Fluor 594-conjugated goat-anti rabbit IgG secondary antibody (diluted 1:250, Invitrogen, Grand Island, NY, USA) for two hours at room temperature. We put the sample on the slides and then, the blinded tester counted the number of neutrophils in the hippocampus from both hemispheres under fluorescent microscope. The total average number of neutrophils from the hippocampus region was used for statistical analysis.

### 2.12. Data Analysis

Data are displayed as the mean ± S.E.M. Statistical significance between experimental groups was measured by analysis of variance (ANOVA) in accordance with the Bonferroni post-hoc test. Differences were considered statistically significant at *p* < 0.05.

## 3. Results

### 3.1. DCA Inhibits PDK2 after Hypoglycemia

**Figure 1:**
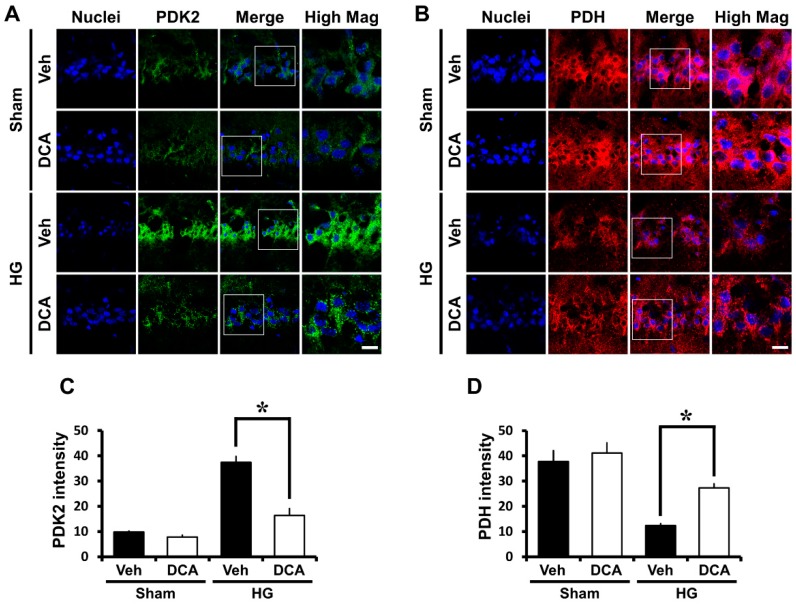
Effects of sodium dichloroacetate (DCA) on hypoglycemia-induced pyruvate dehydrogenase kinase 2 (PDK2) activation and pyruvate dehydrogenase (PDH) reduction. Fluorescent images show the effect of DCA treatment on PDK2 level after hypoglycemia. (**A**) Difference in PDK2 intensity between vehicle- and DCA-treated groups in the vulnerable CA1 after hypoglycemia. Scale bar = 10μm. (**B**) Difference in PDH intensity between vehicle- and DCA-treated groups in the vulnerable CA1 after hypoglycemia. In normal state, neuronal cells maintain an adequate amount of active PDH, while hypoglycemia causes a significant reduction of active PDH. However, DCA administration recovers PDH activity. Scale bar = 10μm. Bar graph shows quantification of (**C**) PDK2 or (**D**) PDH intensity in CA1 region. Data are mean ± S.E.M., n = 3 from each group. * Significantly different from vehicle treated group, *p* < 0.05.

In previous studies, PDKs were shown to be key regulators in glucose metabolism and to inhibit PDH by phosphorylating the enzyme during brain injury [26,45]. In the present study, we hypothesized that PDKs activation blocks entry of pyruvate into the citrate acid cycle in mitochondria, leading to reduction of ATP formation, and thus, causes neuronal cell death in hypoglycemia. As a result of PDK2 immunostaining, we found that the level of PDK2 significantly increased in the hypoglycemia-induced group compared to the sham group. However, DCA, the inhibitor of PDK2, reduced the level of PDK2, and consequently reduced neuronal cell death (Figure 1A,C).

### 3.2. DCA Increases PDH after Hypoglycemia

To continually maintain life, cells must use glucose as a fuel. This process is mainly controlled by the enzyme PDH, which regulates the entry of glycolytic products into the citric acid cycle by converting pyruvate into acetyl-CoA in the mitochondria [46]. PDH is usually suppressed by PDH- induced phosphorylation [47]. According to previous studies, pyruvate dehydrogenase activity is reduced in neurodegenerative brain diseases such as Huntington and Alzheimer’s [48,49]. Based on these previous results, we conducted PDH staining to investigate if active PDH is similarly inhibited after hypoglycemia and to determine if this results in the loss of neuronal cells. We discovered that the level of PDH significantly decreased in the hippocampal CA1 region in the hypoglycemia-induced group compared with the sham group. In the present study we found that the administration of DCA increased the level of PDH and reduced hypoglycemia-induced neuronal death (Figure 1B,D).

### 3.3. DCA Decreases Neuronal Death after Hypoglycemia

**Figure 2:**
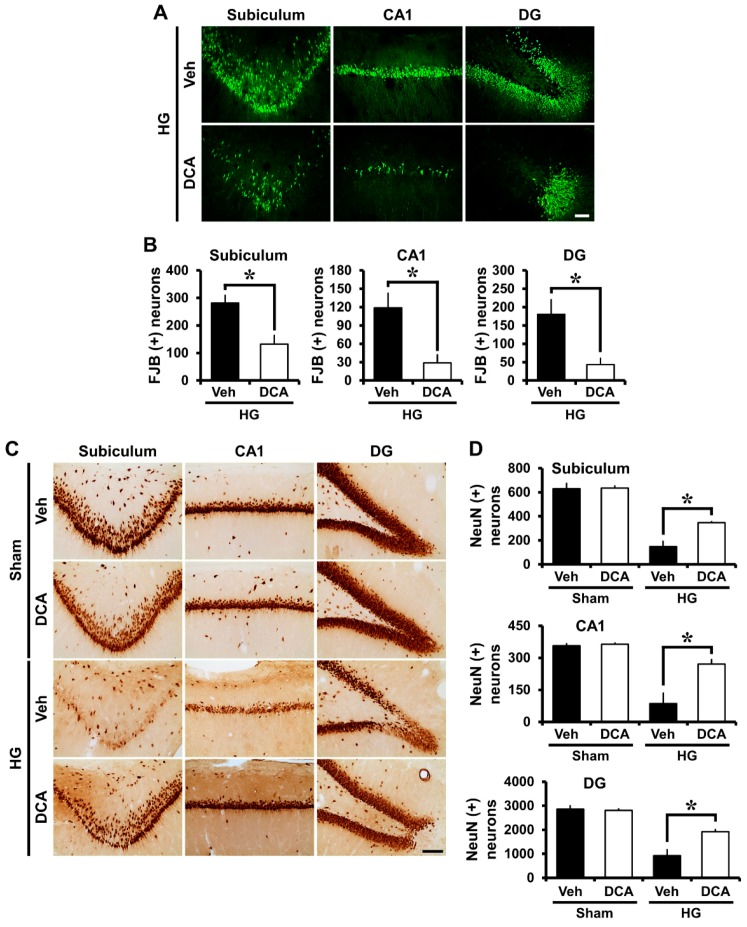
Effects of DCA on hypoglycemia-induced neuronal death. Hypoglycemia resulted in neuronal death in the subiculum (sub), CA1, and dentate gyrus (DG) of hippocampus one week after hypoglycemia. (**A**) Fluoro-Jade B (FJB) positive neuronal cells are observed in the CA1, subiculum, and DG one week after hypoglycemia. Scale bar = 50 μm. (**B**) The number of FJB (+) cells were significantly lower in the DCA-treated group than that in the control group after insult. Data are mean ± S.E.M., n = 8 from each hypoglycemia group. (**C**) NeuN (+) cells show live neurons in the hippocampal subiculum, CA1 and DG. Scale bar = 100 μm. (**D**) The number of NeuN (+) cells were considerably more increased in the DCA-treated group than in the control group after insult. Data are mean ± S.E.M., n = 3 for each sham group and hypoglycemia group. * Significantly different from vehicle treated group, *p* < 0.05.

Severe neuronal death is caused by hypoglycemia and subsequent glucose reperfusion when estimated at seven days after injury [4]. After hypoglycemia, we performed NeuN staining in order to confirm the number of surviving neurons, and also Fluoro-Jade B (FJB) staining in order to detect degenerating neurons in the hippocampal subiculum (sub), CA1 and dentate gyrus (DG). First, Fluoro-Jade B staining, a selective marker of degenerating neurons, exposed broad hippocampal neuronal cell death in the subiculum (sub), CA1, and dentate gyrus (DG) after insult. Rats treated with DCA (100 mg/kg, i.v., two days) displayed a significant reduction in hippocampal neuronal death after hypoglycemia (Figure 2A). As demonstrated in Figure 2B, rats given DCA showed reduced FJB (+) neurons in the subiculum, CA1, and DG by 53%, 76%, and 76%, respectively, compared with rats given only saline plus glucose. Moreover, sham-operated groups showed live neurons in the hippocampal subiculum, CA1 and dentate gyrus via NeuN staining. There were no significant differences in the NeuN (+) cell numbers between vehicle- and DCA- treated group. Compared to the sham group, the number of surviving neurons was significantly decreased at 1 week after hypoglycemia. However, the number of surviving neurons in the DCA-treated group was significantly higher than in the vehicle-treated group (Figure 2C). As shown in Figure 2D, rats given DCA showed increased NeuN (+) neurons in the subiculum, CA1, and DG by 35%, 51%, and 35%, respectively, compared with rats given only saline plus glucose.

### 3.4. DCA Reduces Hypoglycemia-Induced Oxidative Injury

**Figure 3:**
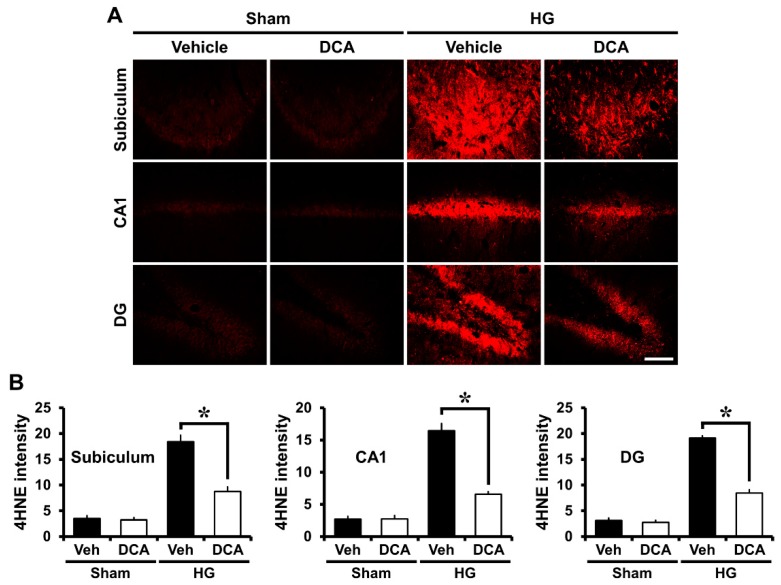
Administration of DCA decreases oxidative injury after hypoglycemia. Neuronal oxidative injury was determined by 4-hydroxy-2-nonenal (4HNE) (red color) staining in the hippocampal regions seven days after hypoglycemia. (**A**) Sham-operated groups rarely displayed the 4HNE signal in the hippocampus. DCA decreased the intensity of 4HNE fluorescence in the hippocampus compared with the saline-treated group after hypoglycemia. Scale bar = 100 μm. (**B**) 4HNE fluorescence intensity in the hippocampus. The fluorescence intensity indicates a significant gap between saline- and DCA-treated groups. Data are mean ± S.E.M., n = 5 for each sham group. n = 8 for each hypoglycemia group. * Significantly different from vehicle treated group, *p* < 0.05.

Mitochondria are the main sources of ROS production in many disorders such as ischemia, traumatic brain injury, or seizure [50]. Mitochondria taken from brain during hypoglycemia exhibit increased capacity to produce ROS [51]. Our previous study suggested that ROS can be produced by NADPH oxidase activation after hypoglycemia, so the production of ROS causes oxidative stress-induced neuronal cell death in the hippocampus [4]. We conducted 4-hydroxynonenal (4HNE) staining to estimate the degree of oxidative damage after hypoglycemia. Rat brains were immunohistochemically stained with a 4HNE antibody one week after injury to reveal whether oxidative stress had occurred in the hippocampal neurons and whether DCA can reduce this negative effect on neurons. In the sham-operated group, both saline controls and DCA-treated rats demonstrated no difference in the fluorescence intensity of the 4HNE staining in the subiculum, CA1, and DG. In the hypoglycemia-operated group, 4HNE fluorescence intensity increased in the hippocampus region of the saline only group and but decreased in the DCA-treated group after hypoglycemia (Figure 3A). As demonstrated in Figure 3B, group treated with DCA and then 25% glucose showed an approximately 53% reduction in the intensity of 4HNE in the subiculum, 60% in the CA1, and 56% in the DG compared with the group provided only glucose (Figure 3B). Therefore, this result indicates that DCA can reduce oxidative injury, and thus spare hippocampal neurons from hypoglycemic insult.

### 3.5. DCA Reduces Hypoglycemia-Induced Microglia and Astrocyte Activation

**Figure 4:**
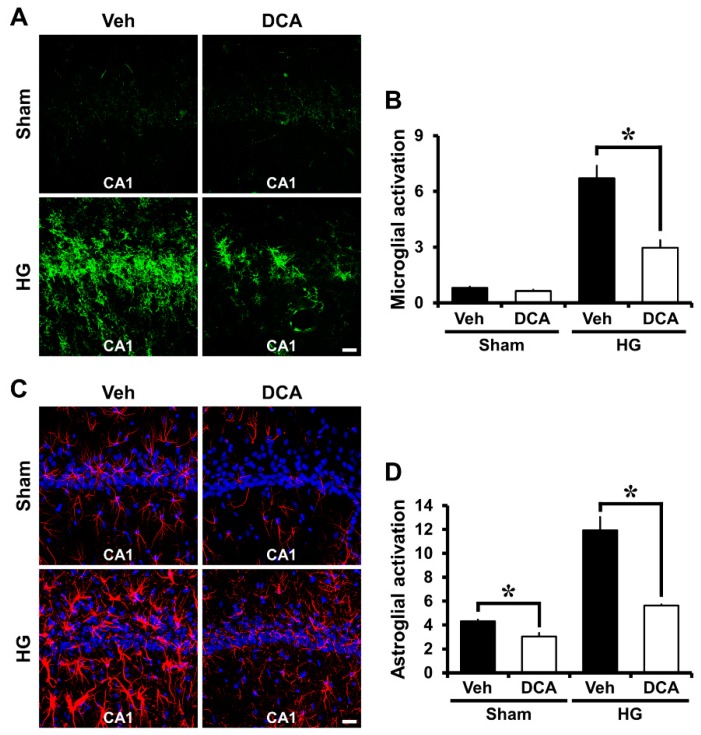
Administration of DCA decreases microglia and astrocyte activation after hypoglycemia. To detect microglia activation, we conducted CD11b staining. Hypoglycemia-induced microglia activation triggers an immune response. CA1 is vulnerable to damage by activated microglia. **(A)** The green fluorescence (CD11b staining) shows activated microglia in the hippocampal CA1 area. Sham-operated group shows almost no microglial activation. However, after hypoglycemia, microglia cell number, intensity, and morphology were greatly enhanced in the vehicle-treated group compared to the DCA-treated group. Scale bar = 20 μm. Data are mean ± S.E.M., n = 5 for each sham group, n = 8 for each hypoglycemia group. (**B**) The grade of microglia activation in the CA1. (**C**) The red fluorescence (GFAP staining) shows activated astrocyte in the hippocampal CA1 area. The sham-operated group shows almost no astrocyte activation. However, after insult, DCA administration prevented astrocyte activation in the CA1. Scale bar = 20 μm. (**D**) The graph represents the intensity of activated astrocytes in the CA1 region. Data are mean ± S.E.M., n = 3 for each sham group and hypoglycemia group. * Significantly different from vehicle treated group, *p* < 0.05.

Another study showed that hypoglycemia induces microglia and astrocyte activation in the cerebral cortex and the hippocampus [52]. Severe hypoglycemia was induced in rats and then brains were harvested one week after insult. We therefore evaluated the degree of microglia and astrocyte activation between four groups: Sham (Vehicle, DCA) and Hypoglycemia (Vehicle, DCA). As a result of the staining, sham-operated groups showed resting microglia and small astrocytes. Also, activated astrocytes have the potential to be harmful because they can express NOS and produce neurotoxic NO [53,54]. Compared with the sham-operated groups, microglia and astrocyte activation in the CA1 increased by approximately 66% and 60%, respectively, in the hypoglycemia-operated group. However, after insult, microglia and astrocyte activation decreased around 56% and 46% in the DCA-treated group compared to the saline-treated group. Therefore, administration of DCA can decrease microglia and astrocyte activation associated with inflammation (Figure 4A–D).

### 3.6. DCA Prevents Hypoglycemia-Induced Blood-Brain Barrier (BBB) Disruption

**Figure 5:**
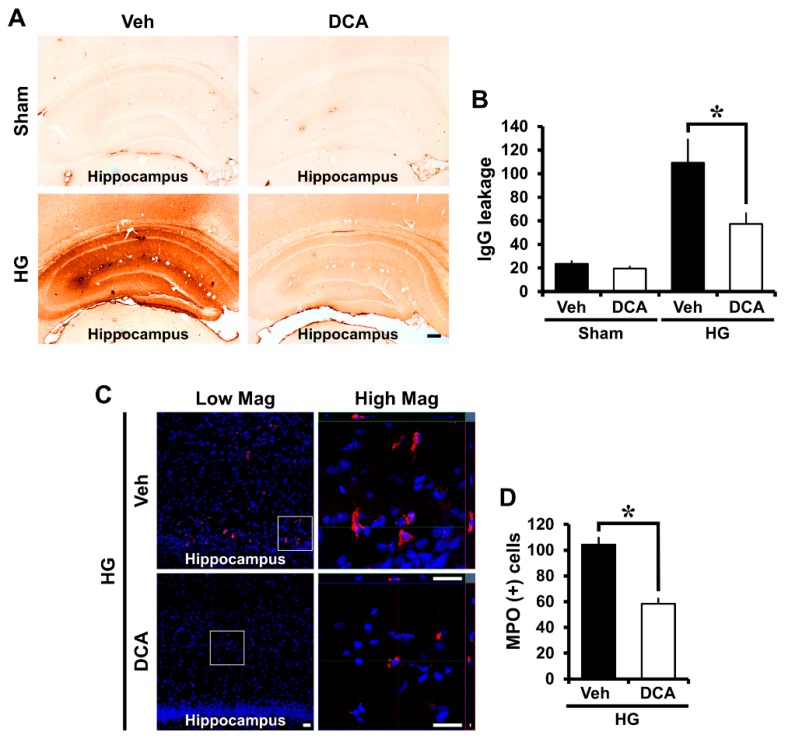
Administration of DCA decreases blood-brain barrier (BBB) breakdown and an influx of neutrophils after hypoglycemia. (**A**) Whereas sham-operated group had little leakage of IgG, the hypoglycemia group had a large quantity of IgG leakage when the BBB broke down. However, the DCA–treated group had significantly reduced IgG leakage. Scale bar = 200 μm (4×). (**B**) The quantification of IgG leakage in the whole hippocampus. Data are mean ± S.E.M., n = 5 for each sham group, n = 8 for each hypoglycemia group. Data are mean ± S.E.M., n = 5 for each sham group, n = 8 for each hypoglycemia group. (**C**) Representative immunofluorescence images show expression of the neutrophil marker MPO in the cortex and hippocampus. DCA prevent Scale bar = 20 μm. (**D**) The bar graph indicates the number of MPO (+) cells in the cortex and hippocampus. Data are mean ± S.E.M., n = 3 for each sham group and hypoglycemia group. * Significantly different from vehicle treated group, *p* < 0.05.

Many other studies reported that blood-brain barrier (BBB) disruption occurs under brain injury conditions such as ischemia, traumatic brain injury, and seizure [55,56,57]. In severe hypoglycemia, BBB is destroyed as early critical events, because glucose deprivation destroys endothelial cells that make up BBB [58,59,60]. To determine disruption of the BBB, we conducted IgG staining by detecting the degree of extravasation of serum immunoglobulin G (IgG) with a previously described method [43] and myeloperoxidase (MPO) staining to look for an influx of neutrophils after hypoglycemia. Firstly, IgG produced coronas with a concentration gradient around the blood vessels in the hippocampus area, and IgG staining was observed in broad areas in the brain due to hypoglycemia. Generally, in the sham-operated group, little leakage of IgG occurred, but the group experiencing hypoglycemic status experienced IgG leakage from damaged vessels. Figure 5 shows that a significant difference was found in the concentration gradient of IgG leakage between the sham and hypoglycemia-induced groups. IgG leakage via BBB disruption was apparent in the vehicle-treated group following hypoglycemia. However, administration of DCA significantly decreased hypoglycemia-induced IgG leakage through BBB disruption in the hippocampus (Figure 5A,B). In additionally, we conducted MPO staining to check BBB permeability increased by the destruction of BBB after hypoglycemia. The increase of BBB permeability causes an influx of neutrophils, and neutrophils are identified through MPO staining. As a result of the MPO staining, sham-operated groups did not show MPO (+) cells. After hypoglycemia, compared with the sham-operated groups, MPO (+) cells were found to be significantly increased, but DCA administration reduced the infiltration of neutrophils in the hippocampus (Figure 5C,D).

## 4. Discussion

Previous studies showed that severe hypoglycemia can cause seizure, unconsciousness, and neuronal death as extreme end-points [8,38,39]. The mechanisms of hypoglycemia-induced neuronal cell death are still unclear. Our previous study demonstrated that neuronal cell death induced by hypoglycemia is caused not only by the low glucose level itself but also by glucose reperfusion [37]. Hypoglycemia causes excessive vesicular zinc release, which leads to NADPH oxidase activation and causes neurotoxicity [4,9]. Zinc influx into neurons after hypoglycemia can lead to mitochondrial dysfunction, resulting in reduced ATP levels in the brain [61]. Therefore, we investigated whether treatment with DCA, an activator of ATP formation, has neuroprotective effects in the hippocampus after severe hypoglycemia.

The ATP required for neurological function in the brain is predominantly generated by glucose oxidation (GO) and pyruvate oxidation (PO) in mitochondria [62] and it is closely related to hypoglycemic state. Under physiological conditions, formation of pyruvate increase rates of glycolysis and enhances glucose oxidation by way of activation of PDH, which converts pyruvate into Acetyl-CoA. However, under pathophysiological conditions such as hypoglycemia (HG) and ischemia-reperfusion (IR), it decreases the rates of glucose oxidation due to mitochondrial dysfunction [62,63]. Depressed glucose oxidation can lead to neuronal cell death [49,64].

Dysfunction of mitochondrial metabolism is central to the pathological results following brain injury, such as traumatic brain injury or ischemia [65,66]. Previous studies reported that PDH is important in altered brain energy metabolism in diverse brain injuries [67,68,69]. Traumatic brain injury is reported to enhance the expression of PDK and phosphorylate PDH, leading to inhibition of PDH-regulated glucose metabolism [45]. Bowker-Kinley et al. mentioned that when they looked at levels of PDK mRNA, PDK1 (heart), PDK3 (testis), PDK4 (skeletal muscle and heart) was found in certain tissues, whereas PDK2 was found in all tissues. Also, they indicated that in the brain, for example, PDK activity corresponds primarily to the isoenzyme PDK2. In addition, they showed that DCA, a structural analog of pyruvate, attaches to the pyruvate-binding site, resulting in inhibition of PDKs with the order of inhibition being PDK2 > PDK1~PDK4 >> PDK3 [23]. In the present study, we confirmed that hypoglycemic insult increased the level of PDK2 in the hippocampus and thus reduced PDH, causing inhibition of glucose metabolism. However, administration of DCA decreased PDK2 levels in the hippocampus.

After several brain insults, mitochondrial damage induces excessive PDK activation, which restricts ATP formation and results in more severe neuronal death [26,45]. In the case of severe hypoglycemic animal models, neuronal death occurs, and given the result, mitochondrial dysfunction occurs in both the hypoglycemic state itself and also during the hyperglycemic state when glucose is reperfused [51,70,71,72]. DCA is known to be neurotoxic when high doses, above 500 mg/kg, are used, or if 300 mg/kg is administered for more than 10 weeks [73,74]. However, many previous animal studies have confirmed the beneficial effects of administering DCA at dose of 50 to 200 mg/kg [75,76,77,78,79], and our previous study also confirmed that treatment with a dose of 100 mg/kg after ischemia reduced neuronal death [26]. According to the paper by Stacpoole, it was noted that DCA was rapidly absorbed following treatment, crosses the blood-brain barrier and activates PDH within a few minutes [80].

**Figure 6:**
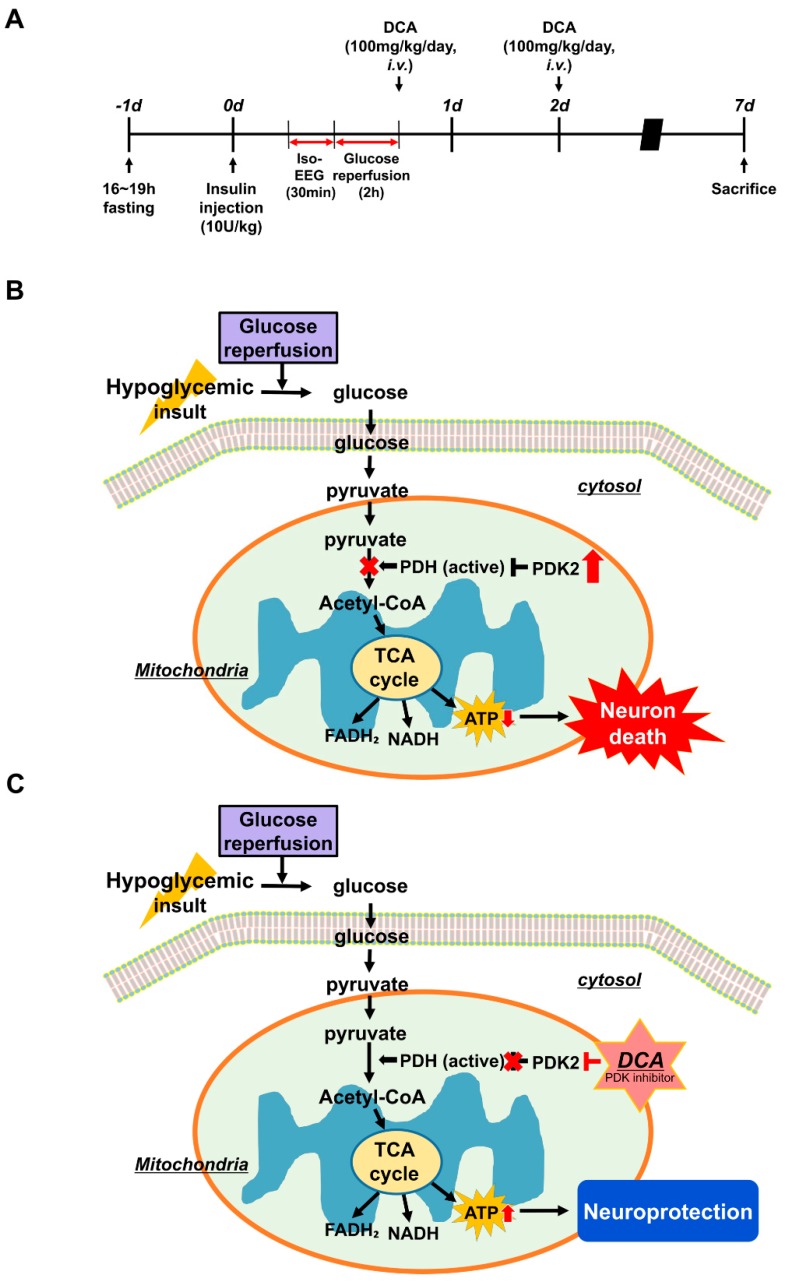
Possible association of PDK2, PDH, DCA, and neuronal death under hypoglycemic conditions. This schematic illustration shows the effects of DCA on the process of hypoglycemia-induced hippocampal neuronal death. (**A**) Experimental timeline. (**B**) Neuronal cell death mechanism caused by hypoglycemia: (1) After hypoglycemia, mitochondria disruption occurs and then PDK2 increase abnormally (2) An abnormally increased PDK2 inhibits the activity of PDH by phosphating PDH (3) The conversion from pyruvate to acetyl-CoA is unsuccessful due to the inhibition of PDH (4) The rate of synthesis of ATP through the TCA cycle significantly reduces. When these conditions dominate, neuronal death is more likely to occur. (**C**) Effects of DCA on hypoglycemia-induced neuronal cell death: administration of DCA can inhibit PDK2 and thus prevent hippocampal neuronal death after hypoglycemia.

In the present study, we found that DCA, a PDK inhibitor, significantly reduces the number of degenerating neurons after hypoglycemia. FJB staining demonstrated a decline in the number of degenerating neurons in the hippocampus in the DCA-treated group compared to the vehicle-treated group after hypoglycemia. This trend correlated with the NeuN staining, which indicated an increase of surviving neurons in the DCA group. This result suggests that DCA increases PDH levels by inhibiting PDK2, which may result in increased ATP synthesis, reducing neuronal cell death in the subiculum, CA1 and dentate gyrus of hippocampus compared with the vehicle-treated group, after hypoglycemia (Figure 6).

D’Alessandro et al. stated that mitochondrial impairment induced by the reduction of the glucose-derived pyruvate results in decreased glutathione synthesis [39,81]. Glutathione plays an important role in the cells, which reduces the oxidative stress by acting as an antioxidant. This synthesis of glutathione requires glycine, glutamate and cysteine, and consumes ATP [82]. Mitochondrial dysfunction after hypoglycemic insult contributes to oxidative stress by activating NADPH oxidase, leading to ROS formation, which is heavily associated with neurodegenerative diseases [4,9,83]. Therefore, we conducted 4HNE staining to confirm whether DCA decreases oxidative stress in the hippocampus. We found that DCA administration successfully reduced oxidative injury in the hippocampus compared to the vehicle-treated group after hypoglycemia. Therefore, we assumed that the administration of DCA increases the level of ATP, and this has resulted in the reduction of the oxidative stress due to the efficient supply of ATP for glutathione formation.

Neural or immune cells participating in neuroinflammation experience metabolic changes including a glycolytic metabolic shift. The altered glycolytic metabolism leads to promotion or inhibition of neuroinflammation [84,85]. PDKs have been reported to control functional polarization of macrophages [86]. Macrophage polarization toward the pro-inflammatory M1 phenotype is usually followed by a cellular metabolic change from oxidative phosphorylation to aerobic glycolysis, as well as nitric oxide (NO) production [86]. Likewise, NO produced by brain diseases suppresses enzyme activity in PDH [87]. Jha et al. suggested that PDK2/4 play vital roles in the inflammatory infiltration of immune cells, in the induction of the pro-inflammatory macrophages, and in inhibition of the anti-inflammatory phenotype of peripheral macrophages [88]. Microglia and astrocyte can be activated immediately during brain injury and induce pro-inflammatory molecules such as tumor necrosis factor-α (TNF-α) or NO [89,90]. Therefore, brain inflammation has been considered to be a potential target in treating brain diseases for several years, and various approaches have been implemented to suppress disease-induced brain inflammation [91,92,93]. The selective vulnerability of CA1 hippocampus is very well studied in rodents and CA1 is known to have the highest susceptibility to damaging conditions [94,95,96]. We performed CD11b and GFAP staining, which is an inflammatory marker, to confirm the neuroprotective effects of DCA on inflammation induced by microglia and astrocyte activation after hypoglycemia. Thus, we found that DCA reduced microglia and astrocyte activation by inhibiting PDK2 in the hippocampal CA1 region after hypoglycemia.

BBB disruption was assessed on the basis of IgG extravasation [43] and is related to an influx of neutrophils after brain insult [97]. Generally, IgG concentration and an infiltration of neutrophils in the brain is rare because it exists in the blood vessels when undamaged. However, under pathological conditions, IgG leakage and neutrophils influx occur as the BBB is disrupted by brain injuries such as seizure, ischemia, or traumatic brain injury [55,98,99,100]. Doll et al. reported that mitochondria play an important role in the opening of the BBB [101]. If mitochondria are destroyed by lack of oxygen or glucose, ATP production decreases in the endothelial cells surrounding the blood vessels in the brain, resulting in the disruption of the BBB, which exacerbates brain injury [102]. Mitochondrial dysfunction also causes the collapse of the BBB because it further reduces the rate of ATP synthesis [101,103]. Therefore, we conducted IgG and MPO staining to confirm whether DCA prevents the BBB breakdown by increasing the formation of ATP. Here, we found that DCA decreased IgG leakage in the whole brain and the number of MPO (+) cells in the hippocampus after hypoglycemia. This result demonstrates that DCA has the capability to prevent BBB disruption by enhancing ATP production in brain endothelial cells after hypoglycemia.

In conclusion, we found that DCA treatment can alleviate hippocampal neuronal death by inhibiting PDK activity following hypoglycemia. It is unclear exactly how PDK2 is increased by hypoglycemia/glucose reperfusion. We speculate that a complex signaling pathway may be generated by the hypoglycemic insult inside mitochondria. Therefore, our findings suggest that DCA may be a vital therapeutic tool for preventing neuronal death induced by hypoglycemia.
